# Proteomic Analysis of *S*-Nitrosation Sites During Somatic Embryogenesis in Brazilian Pine, *Araucaria angustifolia* (Bertol.) Kuntze

**DOI:** 10.3389/fpls.2022.902068

**Published:** 2022-06-30

**Authors:** Alexandre Junio Borges Araujo, Giovanni Victorio Cerruti, Rafael Zuccarelli, Marta Rodriguez Ruiz, Luciano Freschi, Ratna Singh, Bruno Maria Moerschbacher, Eny Iochevet Segal Floh, André Luis Wendt dos Santos

**Affiliations:** ^1^Departamento de Botânica, Instituto de Biociências, Universidade de São Paulo, São Paulo, Brazil; ^2^Department of Plant Biology and Biotechnology, WWU Münster, Münster, Germany

**Keywords:** nitric oxide, nitrosoproteomic analysis, nitrosative/oxidative stress, conifers, embryogenesis

## Abstract

Cysteine *S*-nitrosation is a redox-based post-translational modification that mediates nitric oxide (NO) regulation of various aspects of plant growth, development and stress responses. Despite its importance, studies exploring protein signaling pathways that are regulated by *S*-nitrosation during somatic embryogenesis have not been performed. In the present study, endogenous cysteine *S*-nitrosation site and *S*-nitrosated proteins were identified by iodo-TMT labeling during somatic embryogenesis in Brazilian pine, an endangered native conifer of South America. In addition, endogenous –S-nitrosothiol (SNO) levels and S-nitrosoglutathione reductase (GSNOR) activity were determined in cell lines with contrasting embryogenic potential. Overall, we identified an array of proteins associated with a large variety of biological processes and molecular functions with some of them already described as important for somatic embryogenesis (Class IV chitinase, pyruvate dehydrogenase E1 and dehydroascorbate reductase). In total, our *S*-nitrosoproteome analyses identified 18 endogenously *S*-nitrosated proteins and 50 *in vitro* S-nitrosated proteins (after GSNO treatment) during cell culture proliferation and embryo development. Furthermore, SNO levels and GSNOR activity were increased during embryo formation. These findings expand our understanding of the Brazilian pine proteome and shed novel insights into the potential use of pharmacological manipulation of NO levels by using NO inhibitors and donors during somatic embryogenesis.

## Introduction

Somatic embryogenesis (SE) may be defined as the developmental restructuring of somatic cells toward the embryogenic pathway, being the basis of cellular totipotency in higher plants (Elbl et al., 2015; Aguilar-Hernández and Loyola-Vargas, 2018). SE is a highly useful tool in plant biotechnology as it allows for efficient mass clonal propagation of elite plants, *ex situ* conservation of vulnerable and endangered species, as well as the study of the genetic and physiological changes associated with embryo formation (Elbl et al., 2015; Aguilar-Hernández and Loyola-Vargas, 2018; Botini et al., 2021). In general, most of the stimuli needed to trigger the embryogenic route are obtained by the application of physical stresses and/or exogenous plant growth regulators (García-Mendiguren et al., 2016; Elhiti et al., 2018). In conifers, osmotic stress and/or high non-physiological concentrations of ABA have been used to promote the differentiation of proembryogenic masses (PEMs) into mature somatic embryos (Salaj et al., 2019). However, when cultivated in medium supplemented with maturation promoters, not all embryogenic cell lines are able to differentiate somatic embryos, suggesting that the levels of stress tolerance exhibited by individual cell lines may probably influence the potential for SE (Jo et al., 2014; Zhao et al., 2015; Salo et al., 2016).

Nitric oxide (NO) is a free radical reactive gas acting as a biological mediator in many physiological processes in plants (seed germination, root growth, stomatal closure, and adaptive response to biotic and abiotic stresses) (Nabi et al., 2019). S-nitrosoglutathione reductase (GSNOR), initially identified as glutathione-dependent formaldehyde dehydrogenase, is thought to control the intracellular levels of S-nitrosoglutathione (GSNO), which is considered the major intracellular reservoir and carrier of NO bioactivity (Sakamoto et al., 2002; Leitner et al., 2009). In general, regulatory effects of NO are mediated by protein modifications, including tyrosine nitration, metal nitrosylation, and *S*-nitrosation of cysteine residues. Among them, protein *S*-nitrosation, the reversible covalent attachment of a NO molecule to the thiol side chain of cysteine, can be considered the most relevant due to its effects on protein structure, activity and subcellular localization (Astier et al., 2012; Hu et al., 2015). GSNO levels are known to be in equilibrium with protein S-nitrosothiols (-SNO), and therefore a change in their levels can indirectly affect protein *S*-nitrosation reactions (Sha and Marshall, 2012).

In plants, emerging evidence indicates that GSNOR activity, -SNO endogenous levels and protein *S*-nitrosation may impact biological processes related to growth and development, phytohormone signaling, immune responses, abiotic stress responses and photosynthesis (Jain et al., 2018; Feng et al., 2019). In association with phytohormones (auxin, jasmonic acid, polyamines, and ethylene), NO has been found to play a role during SE (Silveira et al., 2006; Mira et al., 2016), although the occurrence of protein *S*-nitrosation and its physiological and molecular mechanisms affected during stress-induced somatic embryo development remains practically unknown. The development of novel methods for identifying S-nitrosylated proteins such as switch technique using isobaric iodo-TMT reagents, especially when combined with mass spectrometry-based proteomics, has opened new windows of opportunities to identify NO-dependent post-translational modification (PTM) events controlling plant developmental processes (Gould et al., 2013; París et al., 2013).

In the present work, we used a protocol adapted from the Biotin-Switch (Forrester et al., 2009), replacing biotin with the iodo-TMT reagent (iodo-Tandem Mass Tag), to investigate the occurrence of cysteine *S*-nitrosation during somatic embryo formation of Brazilian pine [*Araucaria angustifolia* (Bertol.) Kuntze], an endangered native conifer of South America. Furthermore, we evaluated the endogenous content of –SNO and the activity of GSNOR in cell lines with different embryogenic potential. The stable bond between cysteine (Cys) and iodo-TMT reagent allowed us to sequence the labeled peptides using mass spectrometry, providing both the protein identification and the unequivocal position *S*-nitrosated Cys residues. Using this strategy, we identified proteins associated with a large variety of functional categories such as oxidation-reduction process, defense response, cellular process, carbohydrate metabolic process and translation-related process. In addition, three proteins already described as important for somatic embryogenesis (Class IV chitinase, pyruvate dehydrogenase E1, and dehydroascorbate reductase) were detected as S-nitrosated providing additional evidence for the importance of nitrosative signaling during SE.

## Materials and Methods

### Plant Material

Immature seeds of *A. angustifolia* were collected from three open-pollinated trees grown in the Parque Estadual de Campos do Jordão (22°41.792′ S; 045° 29.393′ W, 1.529 m above sea level) (authorization by Secretaria do Meio Ambiente, Instituto Florestal, in accordance with CARTA COTEC no 066/2014 D139/2013 AP), Campos do Jordão, São Paulo, Brazil. Seeds were mixed to form a pool and then kept at room temperature (up to 48 h) before processing.

### Embryogenic Culture Induction and Proembryogenic Mass Proliferation

Embryogenic culture induction was carried out according to dos Santos et al. (2008), using immature zygotic embryos cultivated in Petri dishes containing BM semi-solid medium (Gupta and Pullman, 1991) free of growth regulators. After 45 days of cultivation in BM medium (dark at 25 ± 2°C), proembryogenic masses (PEMs) induced from immature zygotic embryos were transferred to proliferation medium. For proliferation, PEMs were subcultured every 14 days in MSG semi-solid medium (Becwar et al., 1989) free of growth regulators (dark at 25 ± 2°C).

### Somatic Embryo Formation

To determine the ability to develop somatic embryos, embryogenic cell lines were screened according to Jo et al. (2014), transferring 50 mg (fresh weight) of PEMs to MSG semi-solid culture medium supplemented with 3% (w/v) sucrose, 3% (w/v) sorbitol, 120 μM abscisic acid (ABA), 1% (w/v) Gelrite and subcultured every 8 weeks (dark at 25 ± 2°C). Depending on the response to the maturation medium, embryogenic cell lines were classified as responsive (able to differentiate proembryogenic masses into early somatic embryos) or blocked (not responsive to the formation of somatic embryos) in the medium tested. The maturation tests were conducted annually to certify the classification criteria for selected embryogenic cell lines (3 years evaluation).

### S-Nitrosoglutathione Reductase Activity Assay

S-nitrosoglutathione reductase activity was determined spectrophotometrically as described by Frungillo et al. (2014) with some modifications. Briefly, 500 mg (fresh weight) of embryogenic cell lines were ground with liquid nitrogen in 50 mM phosphate buffer (pH 8.0) and centrifuged at 10,000 × *g* for 10 min at 4°C. The supernatant was incubated at 37°C with 1 mM NADH and 5 mM GSNO (Sigma-Aldrich, San Luis, MO, United States). GSNOR activity was determined by monitoring the consumption of NADH at 340 nm. Protein concentration was measured by the Bradford assay with BSA as the standard (Bradford, 1976).

### Determination of Endogenous – S-Nitrosothiol Levels

Endogenous –SNO levels were quantified according to Mioto et al. (2017). Embryogenic cell lines were ground to powder with liquid nitrogen and extracted in a buffer containing 100 mM Tris–HCl pH 8.0, 100 μM EDTA, 0.1% (v/v) Triton X-100, 10% (v/v) Glycerol, 100 μM neocuproine and 5 mM N-ethylmaleimide (NEM) (500 mg powder: 500 μl buffer). Samples were centrifuged at 16,000 × *g* for 20 min at 4°C, and the supernatants were collected and added to chilled acetone (3:1, chilled acetone: extract volume). Samples were then incubated for 60 min at −20°C and centrifuged again at 16,000 × *g* for 20 min at 4°C. Acetone was discarded, and the pellets were resuspended in the same extraction buffer. An aliquot of 400 μL of extract or standard was mixed with 1.5 μL of a 5 mM diaminorhodamine-4M (DAR-4M) solution in DMSO and then divided into two aliquots of 200 μL. One of these aliquots remained in absolute darkness at room temperature for 5 min, while the other one was transferred into a well in a 0.2 mL PCR plate and placed face-down in a transilluminator (Loccus Biotecnologia, Cotia, Brazil), where it remained for 5 min exposed to UV light (302–312 nm provided by 8 W lamps). Afterward, the volume of both aliquots was adjusted to 1 mL with 50 mM phosphate buffer (pH 7.2), and fluorescence was measured in a PerkinElmer LS-55 spectrofluorometer with excitation and emission recorded at 560 and 575 nm, respectively. The differences between fluorescence in UV-treated and -untreated aliquots were used to estimate the concentration of nitrosylated proteins in the extracts by comparing them to a standard curve of GSNO. Protein concentration was measured by the Bradford assay with BSA as the standard (Bradford, 1976).

### iodo-TMT Specific Labeling, Enrichment and Detection of S-Nitrosated Peptides

iodo-TMT specific labeling, enrichment and detection of S-nitrosated proteins using Western Blot analysis and detection of TMT labeled peptides using LC-MS/MS were performed following the manufacturer’s protocols (available at https://www.thermofisher.com/order/catalog/product/90100).

#### Iodo-TMT Labeling of S-Nitrosated Proteins

For proteomics analyses, PEMs were collected after 14 days of cultivation in MSG proliferation medium, and after 2 and 4 months of somatic embryo maturation. Proteins were extracted from embryogenic cell lines (two biological replicates of 6 g fresh weight each) frozen in liquid nitrogen, and ground to a fine powder in a pre-chilled mortar. Aliquots of 4 g of powder were further ground in 4 ml of HENS buffer [100 mM HEPES (pH 7.8), 1 mM EDTA, 0.1 mM Neocuproine, 1% SDS and 1% (v/v) protease inhibitor cocktail]. Cell debris was removed by centrifugation (10,000 × *g*, 10 min), and protein concentration was determined by BCA assay (Pierce BCA Protein Assay Kit) following the manufacturer’s manual. Protein concentration was adjusted at 1 mg/mL in 1 mL of HENS buffer. For *in vitro S*-nitrosation, proteins were incubated with 500 μM GSNO (Sigma-Aldrich, San Luis, MO, United States) for 30 min at room temperature in the dark. The proteins were incubated with 20 mM methyl methanethiosulfonate (MMTS) at 50°C for 30 min in the dark with frequent vortexing for blocking free cysteine thiols. Residual MMTS was removed by precipitation for 1 h with three volumes of −20°C acetone. Proteins were resuspended in 1 ml of HENS buffer. Labeling with iodo-TMT was achieved by adding 10 μL of iodo-TMT Reagent (Thermo Fisher Scientific, Waltham, MA, United States) dissolved in methanol and 1 M sodium ascorbate for 1 h at 37°C in the dark. The labeling reaction was quenched with 0.5 M dithiothreitol at 37°C for 15 min. Then, proteins were alkylated with 0.5 M iodoacetamide in 1 mL of HENS buffer at 37°C for 1 h.

#### Tryptic Digestion, Peptide Desalting and Anti-TMT Peptide Enrichment

Alkylated samples were precipitated with acetone for 60 min, centrifuged by 10,000 × *g* for 10 min. The pellet was resuspended with 50 mM ammonium bicarbonate containing 20 ng/μL trypsin (Promega, Madison, WI, United States) (ratio of 1:40 w/w, trypsin to protein) and digested overnight at 37°C. The peptides were acidified with 10% (v/v) trifluoroacetic acid (TFA) to final concentration of 0.5% and desalted with 50 mg Waters Sep-Pak tC18 columns and eluted with 70% (v/v) acetonitrile (ACN) and 0.1% (v/v) TFA. For peptide enrichment, the labeled peptides were dried by vacuum centrifugation and resuspended in 120 μL TBS buffer (150 M NaCl, 50 mM Tris, pH 8.0). The peptide samples were incubated with 200 μL anti-TMT resin for 2 h at room temperature with end-over-end rocking. After the supernatant was removed, the resin was washed four times with 200 μL TBS followed by three washing steps with 100 μL HPLC-grade water. Peptides were eluted twice with 400 μL TMT elution buffer (Thermo Fisher Scientific, Waltham, MA, United States), and pooled eluates were dried by vacuum centrifugation.

#### Protein Identification by Liquid Chromatography-Tandem MS

Peptide samples were resuspended in mobile phase A, consisting of water acidified with 0.1% formic acid (FA) and analyzed using an EASY-nLC system (Thermo Fisher Scientific, Waltham, MA, United States) coupled to LTQ-Orbitrap Velos mass spectrometer (Thermo Fisher Scientific, Waltham, MA, United States). The peptides were loaded onto a C18 PicoFrit column (C18 PepMap, 75 μm id × 10 cm, 3.5 μm particle size, 100 Å pore size; New Objective, Ringoes, NJ, United States) and separated with a gradient from 100% mobile phase A (water with 0.1% FA) to 45% phase B (95% Acetonitrile with 0.1% FA) during 100 min, 45–95% in 5 min and 15 min at 95% phase B at a constant flow rate of 300 nL/min. The LTQ-Orbitrap Velos was operated in positive ion mode with data-dependent acquisition in positive ion mode. The three most abundant peptide ions found in full scan were selected for MS/MS and dynamically excluded for a duration of 30 s. The acquisition method is a top 3 × 2, which consists of three HCD events followed by 3 CID events on the same ions selected for the first 3 HCD events. The first scan is a FTMS full scan with resolution of 15000 FWHM and 380–1800 m/z mass range. The three most intense ion found in the full scan were selected and fragmented using HCD with a normalized collision energy of 50, default charge state 3, isolation width 3 m/z and activation time 30 ms. The fragment ions obtained were acquired in the Orbitrap analyzer with a resolution of 7500 FWHM. The same three most intense ions were subsequently fragmented using CID with a normalized collision energy of 35, isolation width 3 m/z and activation time 30 ms. All raw data were accessed in the Xcalibur software (Thermo Fisher Scientific, Waltham, MA, United States) and Proteome Discoverer software version 1.4.0.288 (Thermo Fisher Scientific, Waltham, MA, United States) was used to search the HCD MS/MS spectra against a concatenated database consisting of the 48,246 EST sequences in the *A. angustifolia* transcriptome database (Elbl et al., 2015; dos Santos et al., 2016). The search parameters were set with full trypsin allowing two missed cleavage sites, precursor mass tolerance of 10 ppm and fragment mass tolerance of 0.05 Da. Carbamidomethylation (Cys), Oxidation (Met), acetylation (protein N-terminus), and iodo-TMT (on Cys) were set as dynamic modifications. The resulting peptide hits were filtered for a maximum 1% false discovery rate using the Percolator Node embedded in the Proteome Discoverer software. LC-MS/MS results were obtained from two biological replicates. Only *S*-nitrosated proteins detected in both LC-MS/MS runs (each run corresponding to one biological replicate) were used for bioinformatics analyses. Proteins were reported with at least one matching peptide since, in many cases, only one site in a protein is *S*-nitrosated (Wijasa et al., 2017).

#### Western Blot Analysis

Proteins labeled with the iodo-TMT reagent were resolved on 12% SDS-PAGE gels and transferred onto nitrocellulose membrane using a semi-dry apparatus (Mini-PROTEAN, Bio-Rad), at 100 V for 90 min at 4°C. The membrane was stained with Ponceau S to check equal protein loading. Immunoblots were blocked in 5% non-fat dry milk in TBST buffer for 1 h and were probed with anti-TMT antibody (Thermo Fisher Scientific, Waltham, MA, United States) at 1:1000 dilution for 1 h. For chemiluminescence detection, blots were probed with anti-mouse IgG-HRP conjugate (Pierce) at 1:20,000 dilution and detection was made as suggested by the manufacturer using SuperSignal West Pico Chemiluminescent Substrate (Thermo Fisher Scientific, Waltham, MA, United States).

### Bioinformatic Analysis

Gene Ontology (GO) terms were assigned using PANNZER annotation program (Koskinen et al., 2015)^1^ with default settings. Search for conserved domains was carried out using SMART (Letunic and Bork, 2018)^2^, PROSITE (Sigrist et al., 2013)^3^, and InterPro (Hunter et al., 2009)^4^. Protein domains architecture images were generated with the PROSITE MyDomains-Image Creator tool (Sigrist et al., 2013)^5^. The crystal structure of *Cryptomeria japonica* Chitinase IV (Protein Data Bank code 5H7T) with sequence identity 77.47% was used as template for modeling 3D structure of *A. angustifolia* Chitinase IV. Structure was generated using the Swiss-model (Waterhouse et al., 2018) and USCF Chimera (Pettersen et al., 2004) was applied for the structure optimization and visualization. For protein *in silico* S-nitrosation site prediction, two software packages were tested Group-based Prediction System – GPS-SNO 1.0 (Xue et al., 2010)^6^ and iSNO-AAPair (Xu et al., 2013)^7^. Cysteine disulfide bond partner prediction was carried out using DiANNA 1.1 web server (Ferrè and Clote, 2005)^8^.

### Statistical Analysis

For physiological analyses, PEMs were collected after 14 days of cultivation in MSG proliferation medium, and after 2 and 4 months of somatic embryo maturation. For determination of endogenous -SNO levels four independent biological replicates (obtained from four Petri dishes) were used (each of these biological replicates was run in three technical replicates). For GSNOR activity three independent biological replicates (obtained from three Petri dishes) were used (each of these biological replicates was run in three technical replicates). Student’s *t*-test was used to determine statistically significant differences (*P* < 0.05) in the mean values between pairwise comparisons (cell lines Y1 × B2 during proliferation and cell lines Y1 × B2 during maturation). Analyses were performed using “R” (version 3.5.1, available in http://cran.r-project.org).

## Results

### Somatic Embryo Formation

**FIGURE 1:**
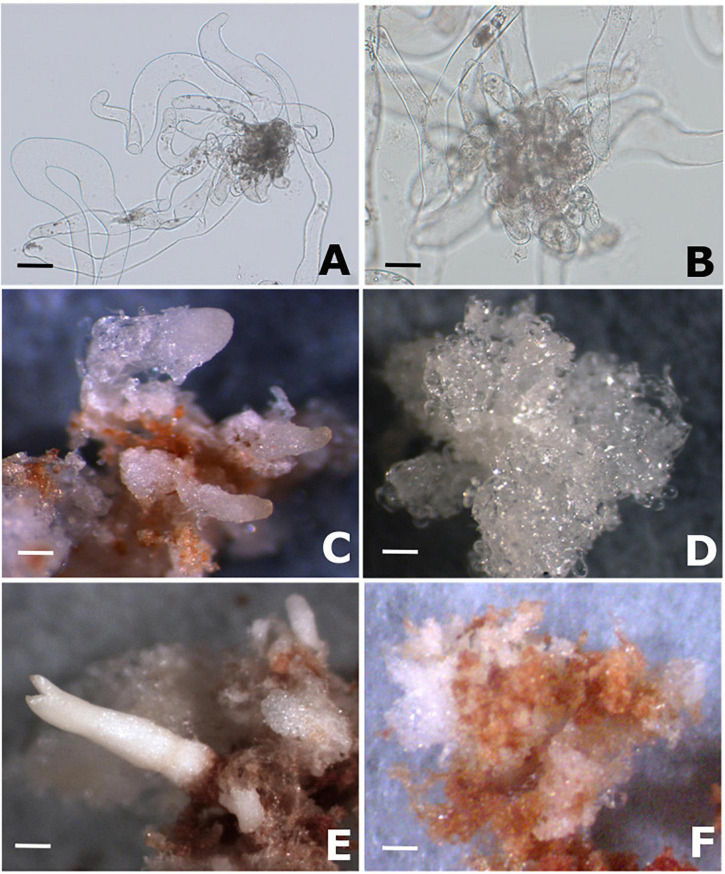
PEMs proliferation and somatic embryo formation in Brazilian pine. **(A)** Morphology of cell line Y1 (responsive to maturation promoters) after 14 of proliferation on semi-solid MSG medium free of growth regulators. **(B)** Morphology of cell line B2 (blocked to the differentiation of somatic embryos) after 14 of proliferation on semi-solid MSG medium free of growth regulators. **(C)** Development of early somatic embryos in cell line Y1 after 2 months of cultivation on semi-solid MSG medium supplemented with 3% sucrose, 3% sorbitol, 120 μM ABA and 1% Gelrite (maturation medium). **(D)** Proembryogenic mass of cell line B2 after 2 months of cultivation on maturation medium. **(E)** Somatic embryo of cotyledonary stage developed in cell line Y1 after 4 months of cultivation on maturation medium. **(F)** Proembryogenic mass of cell line B2 after 4 months of cultivation on maturation medium. Scale bar: 100 μm **(A,B)**; 2000 μm **(C–F)**.

After 4 months of cultivation, two cell lines were selected according to their potential to differentiate somatic embryos. PEMs of cell line Y1 (Figure 1A) demonstrated the ability to differentiated somatic embryos (Figures 1C,E), whereas for PEMs of cell line Figure 1B the development of somatic embryos on the surface of the embryogenic calli was not observed (Figures 1D,F).

### Endogenous – S-Nitrosothiol Contents and S-Nitrosoglutathione Reductase Activity During Proembryogenic Masses Proliferation and Somatic Embryo Formation

**FIGURE 2:**
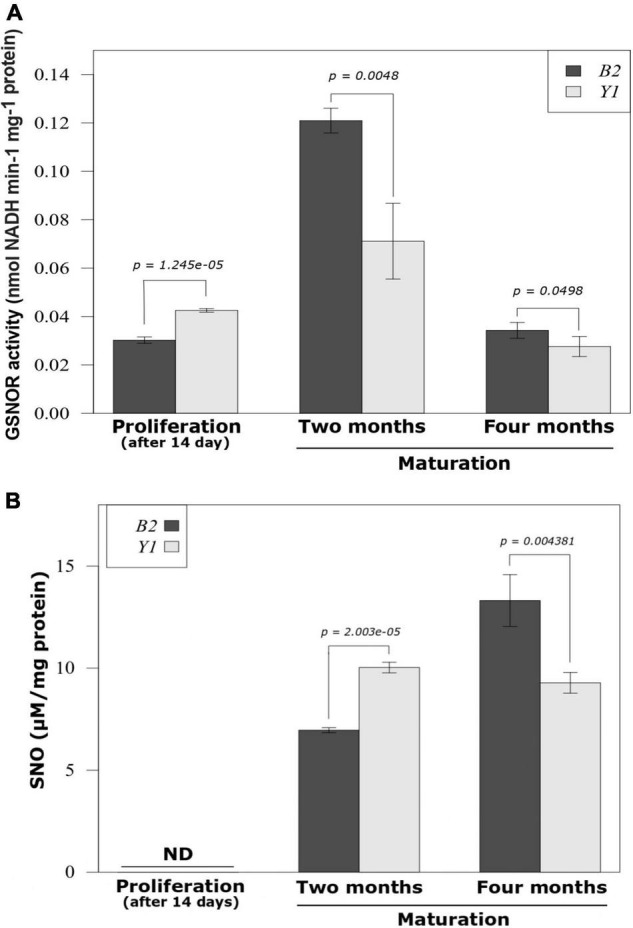
GSNOR activity and endogenous S-nitrosothiol (SNO) level during the proliferation of proembryogenic masses and development of somatic embryos in Brazilian pine. **(A)** S-nitrosoglutathione reductase (GSNOR) activity. **(B)** Endogenous -SNO level. The bars represent the mean and lines indicate the standard error of the mean. Three biological replicates were used for determination of endogenous -SNO levels and four biological replicates were tested for GSNOR activity. Student’s *t*-test was used to determine significant differences in the mean values between pairwise comparisons. B2, cell line blocked to somatic embryo formation; Y1, cell line responsive to somatic embryo formation.

The quantification method used did not allow –SNO detection during the proliferation phase in both cell lines (Figure 2A). Throughout the maturation stage, a marked increase in endogenous -SNO was observed in cell lines B2 and Y1 (Figure 2A). For cell line B2, a progressive increase in the amount of endogenous -SNO was observed (94% between 2 and 4 months of maturation), whereas for cell line Y1, the endogenous SNO levels remained stable during somatic embryo differentiation. Comparatively, after 2 months in maturation medium the amount of endogenous -SNO was significantly higher (*P* < 0.05) in cell line Y1 than that observed for cell line B2 (Figure 2A). However, after 4 months, the endogenous -SNO content was significantly higher (*P* < 0.05) in cell line B2 compared to Y1 (Figure 2A).

For GSNOR activity the values recorded after 14 days of PEMs proliferation were significantly higher (*P* < 0.05) for the Y1 cell line compared to the B2 cell line (Figure 2B). During the maturation phase, the non-responsive cell line (B2) maintained GSNOR activity at significantly higher values (*P* < 0.05) than the cell line responsive to the development of somatic embryos (Y1) (Figure 2B). After 2 months of maturation, GSNOR activity reached the maximum value in both cell lines with an increase in relation to the PEMs proliferation of 400 and 67% in enzyme activity for B2 and Y1, respectively (Figure 2B). However, after 4 months of maturation, GSNOR activity decreased for B2 and Y1 cell lines returning to activity values similar to those observed during PEMs proliferation.

### Brazilian Pine Nitrosoproteome Analysis

In total, 71 peptides (*in vivo* and *in vitro S*-nitrosation) were identified as containing iodo-TMT-labeled cysteine residues (Supplementary Data 1). Of these, 16 peptides were exclusively detected in B2, 32 peptides in Y1 and 23 peptides were shared between embryogenic cell lines Y1 and B2 (Supplementary Data 1). Furthermore, 10 peptides were exclusively detected as endogenously *S*-nitrosated, 48 peptides were exclusively detected as *in vitro S*-nitrosated (after cell extract treatment with GSNO) and 13 peptides were observed in both *in vitro* and *in vivo* samples (Supplementary Data 2). Among them, 68 peptides (95.77%) were identified as containing one *S*-nitrosated cysteine residue and 3 peptides (4.23%) with two *S*-nitrosated cysteine residues. A Chitinase IV (UniProt ID Q5NTA4) identified in cell lines Y1 and B2 after 2 months of maturation showed the highest number of *S*-nitrosated peptides (three in total) (Supplementary Data 2).

Under the conditions tested, GPS SNO 1.0 (medium threshold) presented the best results for prediction of *S*-nitrosation sites, enabling the identification of 28.57% of Cys residues labeled with iodo-TMT reagent (*S*-nitrosation *in vivo*) against 20.68% of those predicted by ISO AAPAIR (Supplementary Data 3). In the test using all *S*-nitrosated Cys residues detected *in vivo* and *in vitro*, ISO AAPAIR detected 20.22% of Cys residues labeled with iodo-TMT reagent while GPS SNO 1.0 predicted 28.84% of the Cys residues (Supplementary Data 3).

### Detection of S-Nitrosated Cysteine Residues During Proembryogenic Masses Proliferation

**FIGURE 3:**
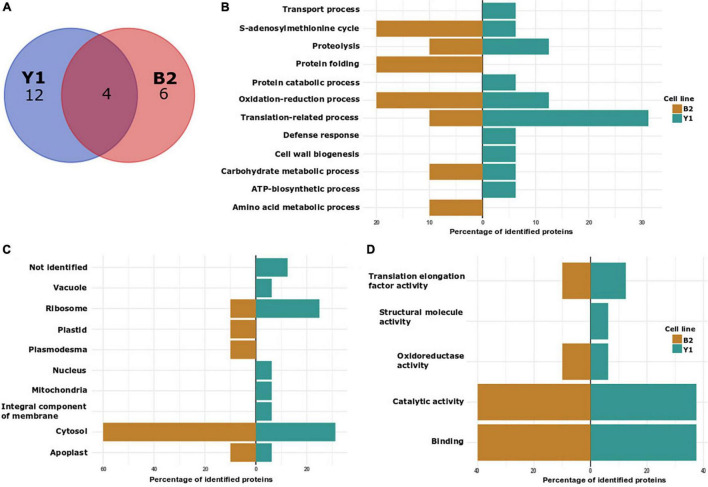
Characterization of *in vitro* S-nitrosated proteins detected during PEMs proliferation in cell lines Y1 and B2 of Brazilian pine. **(A)** VENN diagram depicting the number of identified proteins in cell lines Y1 and B2. **(B)** Biological process classification in GO analysis of proteins detected in cell lines Y1 and B2. **(C)** Cellular component and **(D)** molecular function.

Western blotting analysis using a specific antibody against iodo-TMT reagent did not identify any signs of endogenously *S*-nitrosated proteins (Supplementary Data 4). A weak signal indicating the presence of *S*-nitrosated proteins in cell lines Y1 and B2 was detected only after cell lysate treatment with 500 μM GSNO (*in vitro S*-nitrosation) (Supplementary Data 4). Using the *in vitro S*-nitrosation approach, we identified 19 *S*-nitrosated cysteine residues (corresponding to 16 proteins) in the Y1 cell line, and 10 *S*-nitrosated cysteine residues (corresponding to 10 proteins) in the B2 cell line (Tables 1, 2). Of these, four proteins, namely fructose-bisphosphate aldolase (FBA), adenosylhomocysteinase, malate dehydrogenase and translation elongation factor EFG, were detected in both Y1 and B2 cell lines (Figure 3A). In the set of proteins shared, only (FBA) presented *S*-nitrosated cysteine residues at different sites. In cell line B2, FBA is *S*-nitrosated at Cys74, one position after a tyrosine residue localized at its active site. For cell line Y1, FBA was labeled with iodo-TMT reagent at Cys158, although this position was not identified in the vicinity of an active site or catalytic residue.

**TABLE 1:** Putative *S*-nitrosated proteins and cysteines sites identified by iodo-TMT reagent from GSNO-treated crude extracts of cell line Y1, after 2 weeks of cultivation on proliferation medium MSG (Becwar et al., 1989) supplemented with sucrose and solidified with 0.3% (w/v) Gellan gum.

GO biological process	Accession number	Protein name	Peptide sequence
Translation-related process	A9NTN5	KH type-2 domain-containing protein	GC^134^EVIVSGK
	A0A0C9RRN5	Eukaryotic translation initiation factor 5A	C^58^HFVGIDIFNGK
	A0A200R162	Translation elongation factor EFG	NTGSPTC^267^K
	A0A0C9S3L3	Elongation factor 1-alpha	YYC^87^TVIDAPGHR
	A0A445IE17	Zinc finger protein WIP2	GTQPTAMLRLPCYCCAQGC^37^KNNIDHPR
Proteolysis	A0A2G9I6H7	Cysteine proteinase cathepsin L	AFQFIIQNGGIDNEADYPYEASQGVC^248^K
	A0A2G2XGY5	Subtilisin-like protease	VC^247^DSNGCYSSDIIAAMDR
Defense response	Q9SNX7	Putative intracellular pathogenesis-related protein	AGGGC^114^VSTWTC^120^EYDTLPGVPQDEGK ERVDELDENNFC^82^YK
Transport process	A0A061GV27	MD-2-related lipid recognition domain-containing protein/ML domain-containing protein, putative isoform 1	TTC^102^PVEQGGFTLTNSQSLPGFTPPGAYR
Carbohydrate metabolic process	A0A0D6QRU0	Fructose-bisphosphate aldolase	GLVPLPGSNNESWC^158^QGLDGLASR
Cell wall organization or biogenesis	A0A0C9QTW8	UDP-arabinopyranose mutase	DC^301^TTVQQCYIELSK
Protein catabolic process	A0A1U7YYU8	F-box/LRR-repeat protein 14	LC^415^RC^417^LGVTDIGLKPLVGAHKLQLLR
Oxidation-reduction process	A0A1U7Z1K7	L-ascorbate oxidase	NSWQDGVFGTNC^103^PIPPGR
	A0A0D6R9Z3	Malate dehydrogenase	SQASALEQNAAPDC^125^K
ATP biosynthetic process	A0A0C9SAE7	ATP synthase subunit beta	C^305^ALVYGQMNEPPGAR
S-adenosylmethionine cycle	A0A0C9RQC5	Adenosylhomocysteinase	GETLQEYWWC^240^TER

**TABLE 2:** Putative *S*-nitrosated proteins and cysteines sites identified by iodo-TMT reagent from GSNO-treated crude extracts of cell line B2, after 2 weeks of cultivation on proliferation medium MSG (Becwar et al., 1989) supplemented with sucrose and solidified with 0.3% (w/v) Gellan gum.

GO biological process	Accession number	Protein name	Peptide sequence
Translation-related process	A0A200R162	Translation elongation factor EFG	NTGSPTC^267^K
Protein folding	A0A4D6N4G6	Chaperonin GroEL	C^245^ELENPLILIHEK
	A0A3S3MWD1	IQ motif	SVEYYYTSPFSDSC^19^AVQTR
S-adenosylmethionine cycle/biosynthetic process	A0A0C9RQC5	Adenosylhomocysteinase	GETLQEYWWC^240^TER
	A0A0D6QS11	Methionine adenosyltransferase	VACETC^45^TK
Oxidation-reduction process	A0A0D6R9Z3	Malate dehydrogenase	SQASALEQNAAPDC^125^K
	A0A443P3W9	Aldehyde oxidase/xanthine dehydrogenase	SIPVGVAC^910^ALAAYRLK
Amino acid metabolic process	P51118	Glutamine synthetase cytosolic isozyme 1	GNNILVMCDC^94^YTPAGEPIPTNK
Carbohydrate metabolic process	A0A0D6QRU0	Fructose-bisphosphate aldolase	GILAIDESNATC^74^GKR
Proteolysis	A0A1S3ZXT0	Aspartyl protease family protein 2	SPTC^204^ESDR

In order to elucidate the regulatory roles of identified *in vitro S*-nitrosated proteins, we carried out Gene Ontology (GO) analyses to functionally classify *S*-nitrosated proteins into their biological process, molecular function, and cell component (Figures 3B–D). In cell line Y1, the main GO category affected by GSNO *in vitro* S-nitrosation was translation-related process representing 31.25% of the identified proteins, followed by 12.5% of oxidation-reduction process and 12.5% of proteolysis. Other classes identified were associated with defense response (6.25%), transport process (6.25%), carbohydrate metabolic process (6.25%), cell wall organization or biogenesis (6.25%), protein catabolic process (6.25%), adenosine triphosphate (ATP) biosynthetic process (6.25%), and S-adenosylmethionine cycle (6.25%). For B2 cell line, 20% of the identified proteins were associated with protein folding, 20% with S-adenosylmethionine cycle/biosynthetic process and 20% with oxidation-reduction process. Finally, translation-related process, amino acid metabolism, carbohydrate metabolic process, and proteolysis were annotated, each category containing approximately 10% of total identified proteins. Among the cellular component classification, *in vitro* S-nitrosated proteins were predicted to localize to the cytosol, ribosome and apoplast in both Y1 and B2 cell lines. For the Y1 cell line *in vitro* S-nitrosated proteins were also localized to the nucleus, mitochondria, vacuole and integral component of membrane, whereas for the B2 cell line, proteins were associated with the plasmodesma and chloroplast. In the molecular function classification, catalytic activity and binding were the two main categories annotated for cell lines Y1 and B2.

### Detection of S-Nitrosated Cysteine Residues During Somatic Embryo Formation

Based on the detection of a significant increase in the levels of endogenous -SNO following cultivation in the maturation medium, we decided to evaluate the presence of endogenously *S*-nitrosated proteins (*in vivo S*-nitrosation) during somatic embryo formation. As a control, tests were also carried out treating the cell lysate obtained after 2 and 4 months of maturation with GSNO (*in vitro* S-nitrosation). In total, we identified 14 endogenously S-nitrosated cysteine residues (corresponding to 10 proteins) in cell line Y1 after 2 and 4 months of maturation (Table 3). After treating the Y1 cell lysate with GSNO, we detected 44 *in vitro* S-nitrosated cysteine residues (corresponding to 43 proteins) (Supplementary Data 5). For cell line B2, it was only possible to detect endogenously S-nitrosated cysteine residues after 2 months of maturation (18 sites were identified corresponding to 13 proteins) (Table 4). Finally, after treatment of B2 cell lysates with GSNO, we detected 19 *in vitro* S-nitrosated cysteine residues (corresponding to 19 proteins) after 2 and 4 months of maturation (Supplementary Data 6).

**TABLE 3:** Putative *S*-nitrosated proteins and cysteines sites identified by iodo-TMT reagent from cell line Y1, after 2 and 4 months of cultivation on maturation medium MSG (Becwar et al., 1989) supplemented with sucrose, sorbitol, ABA and solidified with 1% (w/v) Gellan gum.

GO biological process	Accession number	Protein name	Peptide sequence
***After 2 months of maturation***			
Oxidation-reduction process	C7A2A0	Mitochondrial benzaldehyde dehydrogenase	SC^128^VLFR
	A9NV09	Formate dehydrogenase, mitochondrial	C^251^DVVVINMPLSDR
Carbohydrate metabolic process	A0A061E998	Beta-D-xylosidase 4	VNGIPTC^266^ADK
	Q5NTA4	Class IV chitinase	AINSMEC^272^NGGNPSEVSSR
Defense response	Q9SNX7	Putative intracellular pathogenesis-related protein	AGGGC^114^VSTWTC^120^EYDTLPGVPQDEGK KMEAYLLSNPALYC^161^
***After 4 months of maturation***			
Translation-related process	A0A0D6QX01	Ribosomal protein 1	DDPSKPC^41^K
	A0A1U8AGL3	40S Ribosomal protein S11-beta	C^59^PFTGNVSIR
Defense response	Q9SNX7	Putative intracellular pathogenesis-related protein	KMEAYLLSNPALYC^161^
Carbohydrate metabolic process	A0A0D6QYH9	UTP–glucose-1-phosphate uridylyltransferase	LNGGLGTTMGC^101^TGPK
	A0A3S3PBW7	Endo-beta-N-acetylglucosaminidase	QVLLSAAPQC^190^PYPDAHLGR
	Q5NTA4	Class IV chitinase	AINSMEC^272^NGGNPSEVSSR KYPC^191^VSGK TAVWFWMVNSNC^250^HSAITSGK
Oxidation-reduction process	A0A089MX36	Dehydroascorbate reductase	ERGDC^24^PFSQR

**TABLE 4:** Putative *S*-nitrosated proteins and cysteines sites identified by iodo-TMT reagent from cell line B2, after 2 months of cultivation on maturation medium MSG (Becwar et al., 1989) supplemented with sucrose, sorbitol, ABA and solidified with 1% (w/v) Gellan gum.

GO biological process	Accession number	Protein name	Peptide sequence
Carbohydrate metabolic process	A0A0D6QYS8	Pyruvate dehydrogenase E1 component subunit alpha	DC^119^IITAYR
	A0A3S3PBW7	Endo-beta-N-acetylglucosaminidase	QVLLSAAPQC^190^PYPDAHLGR
	Q5NTA4	Class IV chitinase	AINSMEC^272^NGGNPSEVSSR EIAAFFANAAHETGGFC^170^YTEER KYPC^191^VSGK
Translation-related process	A0A1U8AGL3	40S Ribosomal protein S11-beta	C^59^PFTGNVSIR
	A0A0D6QX01	Ribosomal protein 1	DDPSKPC^41^K
	A0A0D6QUM0	60S ribosomal protein L18	AGGEC^55^LTFDQLALR
Oxidation-reduction process	C7A2A0	Mitochondrial benzaldehyde dehydrogenase	AVELAHFALFFNQGQCCC^345^AGSR
	A0A0C9QM69	Glutaredoxin-dependent peroxiredoxin	GVDEIIC^76^ISVNDPFVMR
	A0A0D6R8F1	Succinate–CoA ligase [ADP-forming] subunit beta, mitochondrial	C^361^DIIASGIVNAAK
	A0A089MX36	Dehydroascorbate reductase	ERGDC^24^PFSQR
	A0A0D6R4M7	Cytosolic isocitrate dehydrogenase	NILNGTVFREPILC^116^K
Cellular process	A0A6A2XTS2	Epidermis-specific secreted glycoprotein EP1	C^430^FLTQSLDTLQQLGNTK
	A0A0D6R8S3	Ricin B, lectin domain-containing protein	VYC^115^EANPDFFLAAR
Defense response	Q9SNX7	Putative intracellular pathogenesis-related protein	AGGGC^114^VSTWTC^120^EYDTLPGVPQDEGK ERVDELDENNFC^82^YK

**FIGURE 4:**
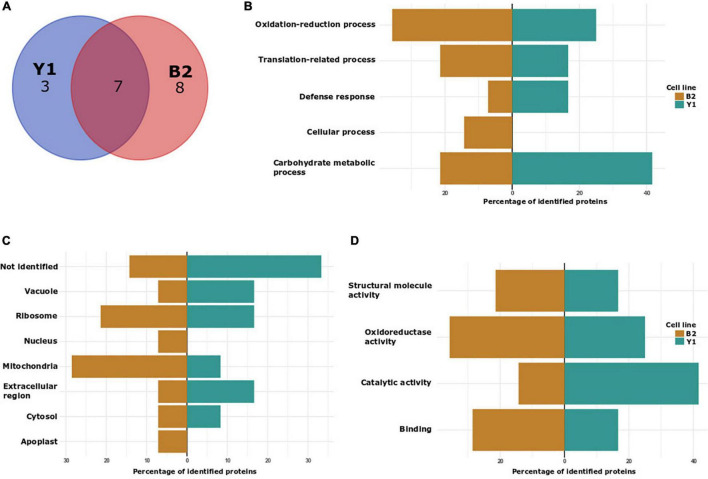
Characterization of *in vivo* S-nitrosated proteins detected during cultivation of cell lines Y1 and B2 of Brazilian pine in medium culture supplemented with maturation promoters (osmotic agents and ABA). **(A)** VENN diagram depicting the number of identified proteins in cell lines Y1 and B2 after 4 months of maturation. **(B)** Biological process classification in GO analysis of proteins detected in cell lines Y1 and B2 after 4 months of maturation. **(C)** Cellular component and **(D)** molecular function.

Six proteins, namely 40S ribosomal protein S11-beta, ribosomal protein 1, endo-beta-N-acetylglucosaminidase, mitochondrial benzaldehyde dehydrogenase, putative intracellular pathogenesis-related protein and Class IV chitinase, were identified as endogenously S-nitrosated in both Y1 and B2 cell lines after callus cultivation in the presence of ABA and osmotic agents (Figure 4A). Among these, mitochondrial benzaldehyde dehydrogenase, putative intracellular pathogenesis-related protein and Class IV chitinase showed peptides labeled with iodo-TMT reagent at different cysteine residues. In cell line B2, mitochondrial benzaldehyde dehydrogenase is S-nitrosated at Cys345, one position before a cysteine residue identified as a catalytic residue and as a NAD(P)-binding site. For putative intracellular pathogenesis-related protein, the peptide AGGGC^114^VSTWTC^120^EYDTLPGVPQDEGK was detected with S-nitrosated Cys residues in positions Cys114 and Cys120 on both Y1 and B2 cell lines, with Cys120 being identified as a hydrophobic ligand binding site. Moreover, in cell line B2, a S-nitrosated Cys residue (Cys82) was also detected in a position before a tyrosine residue (Y) was also identified as a hydrophobic ligand-binding site. For Class IV chitinase, two S-nitrosated peptides AINSMEC^272^NGGNPSEVSSR and KYPC^191^VSGK were detected in both Y1 and B2 cell lines, being Cys272 localized one position after a glutamic acid residue (E) identified as a sugar-binding site. Furthermore, in the B2 cell line, a S-nitrosated Cys residue (Cys170) was detected occupying a position before a tyrosine residue (Y) identified as a sugar-binding site.

Gene ontology (GO) analyses to functionally classify *S*-nitrosated proteins were also carried out for samples obtained *in vivo* (only for proteins detected after 2 months of embryo maturation as after 4 months it was not possible to detect *in vivo* S-nitrosated proteins) (Figures 4B–D) and after GSNO incubation (Supplementary Data 7). Considering biological process categories for endogenously *S*-nitrosated proteins identified in embryogenic cell line Y1, 40% of the proteins were associated with carbohydrate metabolic process, 30% with oxidation-reduction process, 20% with translation-related process and 10% with defense response. In the B2 cell line, after 2 months of PEMs cultivation on the maturation medium, 35.7% of the identified proteins were associated with oxidation-reduction process, 21.4% with translation-related process, 21.4% with carbohydrate metabolic process, 14.3% with cellular response and 7.2% with defense response. In the analysis of cellular components, cytosol, ribosome, mitochondria, vacuole, and extracellular region were annotated to cell lines Y1 and B2, and apoplast and nucleus were exclusively identified in the B2 cell line. In the molecular function classification, catalytic activity and oxidoreductase activity constituted the two main categories annotated to cell line Y1, whilst those associated with oxidoreductase activity and binding were annotated to cell line B2.

### Structural Analysis of a Putative Chitinase IV of Brazilian Pine

**FIGURE 5:**
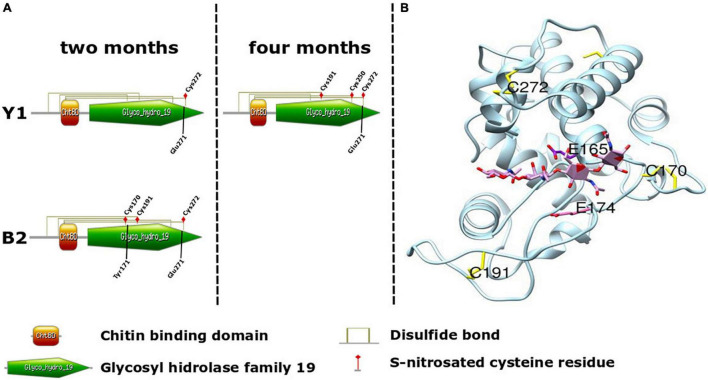
Schematic of Brazilian pine Chitinase IV architecture. **(A)** Domain organization of *Araucaria angustifolia* Chitinase IV. Cysteine residues (Cys) identified as undergoing *in vivo* S-nitrosation during somatic embryo maturation in cell lines Y1 and B2, and involved in the formation of disulfide bond between the Chitin binding domain (orange) and Glycosyl hydrolase family 19 domain (green) are indicated. Tyr171 and Glu271 were identified as enzyme active sites. **(B)** Three-dimensional structures of the *A. angustifolia* Chitinase IV modeled using Swiss-model and USCF-Chimera with the crystal structure of *Cryptomeria japonica* Chitinase IV as template (PDB code: 5H7T). Cysteine residues identified as undergoing *in vivo* S-nitrosation during somatic embryo maturation in cell line Y1 (C191 and C272) and B2 (C170, C191, and C272) and two catalytic residues E165 and E174 are indicated.

Taking into account the importance associated with Class IV chitinase activity during somatic embryogenesis in Angiosperms and Gymnosperms (van Hengel et al., 2001; Dyachok et al., 2002) and for selection of cell lines with high embryogenic potential in Brazilian pine (dos Santos et al., 2008), structural analysis was carried out to map the position of *S*-nitrosation sites to evaluate the possible effects of *S*-nitrosation (Figures 5A,B). To perform this, a putative Brazilian pine Class IV chitinase three-dimensional structure was modeled based on the crystal structure of *Cryptomeria japonica* (PDB code: 5H7T). The *in silico* structural analysis demonstrated that C170 and C272 (Figure 5B) are situated close to the substrate binding site and are in the vicinity of putative catalytic residues E165 and E174. C170 and C272 form disulfide bridges with C121 and C304, respectively. Considering the proximity of the residue C170 with the catalytic residue E174, it is possible that in the absence of disulfide bond the *S*-nitrosation of C170 and C272 may cause a conformational change in the structure of the enzyme and therefore impact substrate binding and catalytic activity. The LC-MS/MS spectra of four representative S-nitrosylated peptides from Class IV chitinase [AINSMEC(7)NGGNPSEVSSR, KYPC(4)VSGK, EIAAFFANAAHETGGFC(17)YTEER and TAVWFWMVNSNC(12)HSAITSGK] were shown in Supplementary Data 8.

## Discussion

### S-Nitrosoglutathione Reductase Activity and Endogenous S-Nitrosothiol Levels During Proembryogenic Masses Proliferation and Somatic Embryo Formation

In our study, we were unable to detect endogenous levels of SNO (Figure 2A) and the presence of endogenously *S*-nitrosated proteins during PEMs proliferation. Moreover, GSNOR activity was significantly higher in cell line Y1 in comparison to B2 during PEMs proliferation (Figure 2B). Despite these results, it is not possible to exclude that in other species, the production of endogenous SNO and the presence of *S*-nitrosated proteins may affect PEMs proliferation and differentiation. Unlike other conifers, PEMs of Brazilian pine are induced and proliferated in the absence of 2,4-D (dos Santos et al., 2008), and the presence of this plant growth regulator can promote protein *S*-nitrosation (Rodríguez-Serrano et al., 2012). After 2 months of cultivation in the presence of ABA and osmotic agents, we observed, regardless of the cell line embryogenic potential, an increase of the SNO contents and GSNOR activity, as well as the existence of endogenously S-nitrosated proteins (Figures 2A,B).

Interestingly, the increase in GSNOR activity during the 2 months of maturation did not reduce SNO production in both cell lines. At the end of the fourth month of maturation, the decrease in GSNOR activity coincided with the increase in endogenous SNO levels in cell line B2, whereas for cell line Y1, the decrease in GSNOR activity did not markedly alter the endogenous SNO contents. Frequently, an inverse correlation between endogenous SNO contents and GSNOR activity is reported in plants submitted to environmental stresses (Simontacchi et al., 2015; Thalineau et al., 2016; Niu et al., 2019). However, depending on the species and type of stress applied, an increase in both GSNOR activity and endogenous SNO content can also be observed (Thalineau et al., 2016). Furthermore, both the enzymatic activity and stability of GSNOR are negatively regulated by *S*-nitrosation, thus preventing GSNO degradation and causing the increase of total -SNOs (Simontacchi et al., 2015; He et al., 2018).

### Qualitative Identification of S-Nitrosated Proteins During Proembryogenic Masses Proliferation and Somatic Embryo Formation

Normally, works reporting protein *S*-nitrosation present data only on *in vivo* or *in vitro S*-nitrosation (after treatment of the protein extract with a NO donor) (Lindermayr et al., 2005; Fares et al., 2011; Gong et al., 2019; Qiu et al., 2019). In our study, we present the results for both approaches (*in vivo* and *in vitro*) using two different Brazilian pine cell lines. Using this strategy, we observed that some of the *S*-nitrosated-Cys residues detected in the *in vivo* analysis were also observed for the *in vitro* fraction (Supplementary Data 9). Similar results were also observed during the comparison between *S*-nitrosated-Cys residues detected in cell lines Y1 and B2. As the approach adopted in the present study was qualitative, it is not possible to determine whether the occurrence of *S*-nitrosation altered the abundance of proteins that were detected. Further studies will have to be performed to determine the occurrence of changes in the ratio between *S*-nitrosated and *S*-nitrosated-free proteoforms. As observed in other plants (Lindermayr et al., 2005; Fares et al., 2011; Gong et al., 2019; Niu et al., 2019; Qiu et al., 2019), *S*-nitrosated proteins identified *in vivo* and *in vitro* during PEMs proliferation and somatic embryo formation in Brazilian pine are involved in a large variety of cellular functions including oxidation-reduction process, translation-related process, carbohydrate metabolic process and proteolysis, and covering different cell components such as ribosome, mitochondria, apoplast, cytosol and vacuole (Figures 3B–D, 4B–D and Supplementary Data 7). With a few exceptions, a similar functional classification of *S*-nitrosated proteins was observed between both cell lines, although the frequency (%) of each category differed markedly during PEMs proliferation and somatic embryo maturation. Considering that endogenously *S*-nitrosated proteins were not detected during PEMs proliferation, cell lysate treatment with GSNO was used to identify potential targets for *S*-nitrosation. It is likely that immediately after the transfer of the PEMs from the proliferation to the maturation medium, the embryogenic cells may be exposed to nitrosative/oxidative stress conditions. According to Silveira et al. (2006) and Durzan (2008), coniferous cells can release nitric oxide quickly and transiently under conditions of stress or mechanical manipulation. Although we could not determine the occurrence of protein *S*-nitrosation during the first days of somatic embryo maturation due to limited source material, we considered that the detection of *S*-nitrosated proteins *in vitro* is relevant for the identification of potential signaling pathways affected by *S*-nitrosation during the initial differentiation of PEMs into early somatic embryos. Below, we discuss selected GO functional categories (translation-related processes, proteolysis and protein stability, ATP biosynthetic process, carbohydrate metabolic process, oxidation-reduction processes and S-adenosylmethionine cycle/biosynthetic process) to gain a better insight into the potential biological significance of S-nitrosated proteins identified during Brazilian pine somatic embryogenesis.

#### Translation-Related Processes

Interestingly, in our study, proteins associated with translation-related processes were the primary biological process observed for *in vitro* S-nitrosated proteins during PEMs proliferation of cell line Y1 (Table 1). Endogenously *S*-nitrosated proteins associated with this GO category were also identified in cell lines Y1 and B2 during cultivation in the presence of ABA and osmotic agents (Tables 3, 4). Somatic embryo development involves gene expression reprogramming, which is regulated by exogenous stimuli produced by plant growth regulators (PGR) or certain stress conditions (Méndez-Hernández et al., 2019). *S*-nitrosation of nuclear protein may affect their subcellular localization or regulate the association with binding partners and consequently alter transcription and/or general nuclear metabolism (Mengel et al., 2013). In plants, several nuclear proteins and zinc-finger-containing transcription factors have been shown to undergo *S*-nitrosation under conditions of nitrosative stress (Sha and Marshall, 2012; Mengel et al., 2013). Altogether, our results demonstrate that translation-related process is potentially affected by *S*-nitrosation, although how this PTM may favor in cell line Y1 the transition from PEMs to early somatic embryo formation needs further experiments.

#### Proteolysis and Protein Stability

In addition to translation-related processes, we detected during PEMs proliferation *in vitro S*-nitrosated proteins related to proteolysis in both cell lines Y1 and B2 (Tables 1, 2) and protein stability in cell line B2 (Table 2). Curiously, proteins associated with protein catabolism or stability were not identified as endogenously *S*-nitrosated during somatic embryo maturation in both cell lines. Proteolytic enzymes and proteins associated with the ubiquitin-proteasome system have been detected in embryogenic and non-embryogenic cell lines, although its role during callus proliferation and somatic embryo formation is still not well understood (Aguilar-Hernández and Loyola-Vargas, 2018). Protein catabolism is an essential determinant for plant growth and development by regulating cell proliferation and cell death (Feng et al., 2007). In conifers, the development of somatic embryos is associated with a reduction in PEMs proliferation after removal of plant growth regulators and by activation of mechanisms of plant cell death (Smertenko and Bozhkov, 2014). NO is known to modulate plant cell death, a process typically observed in the responses of plants faced with stressful situations or that are undergoing a process of differentiation and development (Elhiti et al., 2018).

#### Adenosine Triphosphate Biosynthetic Process

Meanwhile, ATP synthase subunit beta, one of the enzymes responsible for ATP production, was exclusively detected as S-nitrosated in Y1 (Table 1). ATP production and catabolism have been associated with somatic embryo formation and structural reorganization via PCD (Trontin et al., 2016). According to Lindermayr et al. (2005) and Zhang et al. (2017), S-nitrosation reduces the activity of critical enzymes involved in glycolysis and ATP synthase activities, consequently, ATP and other energy sources are diminished after NO treatment. Thus, altogether, both our findings and the published data suggested that S-nitrosation of glycolytic enzymes and ATP production could negatively interfere with somatic embryo formation. However, as observed for ATP synthase, the occurrence of cysteine modification by S-nitrosation may be transient, functioning as a mechanism for protecting cysteine residues against irreversible harmful oxidative modifications (Chang et al., 2014).

#### Carbohydrate Metabolic Process

In our study, we found during PEMs proliferation that Fructose-bisphosphate aldolase (FBA) was S-nitrosated after treatment with GSNO in both cell lines, although at different cysteine residues (Tables 1, 2). FBA is a key plant enzyme involved in glycolysis and gluconeogenesis in the cytoplasm and the Calvin cycle in plastids. Both cytoplasmic and chloroplastic FBA were identified as S-nitrosated in *Arabidopsis thaliana*, *Camellia sinensis* leaves and adventitious roots of *Cucumis sativus* (Lindermayr et al., 2005; Niu et al., 2019; Qiu et al., 2019). Beyond its function in metabolism, FBA plays a role during plant responses to environmental stresses and regulates growth and developmental processes (Lu et al., 2012; Lv et al., 2017). During maturation, we detected only in B2 the presence of endogenously S-nitrosated pyruvate dehydrogenase (E1) (Table 4). In association with dihydrolipoamide acetyltransferase (E2) and lipoamide dehydrogenase (E3), pyruvate dehydrogenase (E1) form a complex responsible for catalyzing the irreversible oxidative decarboxylation of pyruvate to acetyl-CoA and CO_2_. In Arabidopsis, S-nitrosation of dihydrolipoamide inhibits pyruvate dehydrogenase complex activity resulting in polysaccharide-biosynthesis inhibition and monosaccharide accumulation (Zhang et al., 2017). In *Cyclamen persicum* (Lyngved et al., 2008) and *Cyathea delgadii* Sternb. (Domżalska et al., 2017) pyruvate dehydrogenase E1 was found to be upregulated during callus proliferation and differentiation, suggesting a role of this enzyme during SE. In Brazilian pine, a differential accumulation and degradation of starch during the proliferation and maturation phases, respectively, has been associated with cell line responsiveness to somatic embryo formation (Navarro et al., 2017, 2021). Considering the importance of pyruvate dehydrogenase activity in processes related to glycolysis and gluconeogenesis, it will be interesting to determine the impacts of S-nitrosation on the enzyme activity and its implication during the development of somatic embryos.

During somatic embryo maturation in cell lines Y1 and B2, we detected three endogenously S-nitrosated glycosidases, namely endo-β-N-acetylglucosaminidase (ENGases, β-D-xylosidase 4 and chitinase IV) (Tables 3, 4). To the best of our knowledge, despite its importance for the generation of free N-glycans in plants, there is no information if ENGases played a role during embryogenesis or their regulation via S-nitrosation events. For β-D-xylosidase the occurrence of S-nitrosation has been demonstrated during NO-induced adventitious rooting of cucumber (Niu et al., 2019) and stomatal guard cell response to elicitors in Arabidopsis (Lawrence et al., 2020). However, as observed for ENGases, β-D-xylosidase roles during embryogenesis remain to be determined. Chitinases are enzymes hydrolyzing the β-1,4 glycosidic bonds between N-acetylglucosamine residues. All chitinases are grouped into two families (18 and 19) of glycosyl hydrolases and divided according to their primary structure into seven classes (I–VII). Although chitinases usually are produced in plants as a defense response, they are also expressed in healthy plants in an organ-specific and developmentally regulated pattern, suggesting a non-defensive role in plant development (Kasprzewska, 2003; Fischl et al., 2011). During somatic embryogenesis, extracellular chitinases (i.e., specially of class IV) can cleave glycosidic bond of glucosamine and N-acetylglucosamine residues present in the sugar moiety of arabinogalactan proteins, releasing oligosaccharides active as signaling molecules during early somatic embryo formation (van Hengel et al., 2001; Dyachok et al., 2002). The presence of proteins showing chitinolytic activity was observed during zygotic and somatic embryogenesis in Brazilian pine, and class IV was detected as more abundant in cell lines able to respond to ABA and osmotic agents (dos Santos et al., 2006, 2008, 2016). Class IV chitinase was previously reported to be S-nitrosated in poplar leaves exposed to ozone stress (Vanzo et al., 2016), but without the identification of its S-nitrosation site(s). Considering the different S-nitrosated cysteine residues observed during maturation in Y1 and B2 (Figure 5A), and our structural analysis of a putative Chitinase IV of Brazilian pine (Figure 5B) it will be interesting to evaluate in future studies the effects of S-nitrosation in the Chitinase IV capacity for the production of oligosaccharides associated with the cell signaling process.

#### Oxidation-Reduction Processes

Three proteins associated with oxidation-reduction processes (Malate dehydrogenase, L-ascorbate oxidase and aldehyde oxidase/xanthine dehydrogenase) were identified during PEMs proliferation as *in vitro S*-nitrosated (Tables 1, 2). Malate dehydrogenase was identified as sharing the same *S*-nitrosated cysteine residue in both Y1 and B2 cell lines, whereas L-ascorbate oxidase was exclusively identified in Y1 and aldehyde oxidase/xanthine dehydrogenase in B2. Malate dehydrogenase (MDH) is a component of the tricarboxylic acid cycle and catalyzes the interconversion of malate and oxaloacetate coupled to NAD reduction (Qiu et al., 2019). In leaves of *Camellia sinensis*, two subunits of MDH were *S*-nitrosated, suggesting a role of this PTM in regulating enzyme activities associated with pyruvate metabolism (Qiu et al., 2019). NO donors inhibit the activity of MDH, and exposition to abiotic stresses affects its *S*-nitrosation pattern (Jain et al., 2018; Sehrawat et al., 2019). L-ascorbate oxidases (AOs) are glycosylated blue multicopper oxidases of the cupredoxin superfamily responsible for ascorbic acid oxidation with the production of monodehydroascorbate (MDHA) and water. AOs activity regulates the redox state of the apoplastic ascorbate pool, providing an oxidizing environment that prevents cell division and promotes cell expansion (Smirnoff, 2011). Xanthine oxidoreductase and aldehyde oxidase are members of the molybdo-flavoenzyme subfamily, associated with important metabolic roles in purine metabolism, hormone homeostasis and reactive oxygen metabolism (Yesbergenova et al., 2005). In addition, xanthine oxidoreductase is considered a potential enzymatic source of NO in plants (del Rıo et al., 2004). To the best of our knowledge, both L-ascorbate oxidase and xanthine oxidoreductase/aldehyde oxidase have not been previously described f as targets of *S*-nitrosation in plants.

After 2 months of somatic embryo maturation endogenously *S*-nitrosated proteins associated with the oxidation-reduction process were the primary biological process observed in the blocked cell line B2 (35.7% of detected proteins) (Table 4). In contrast, in Y1, three proteins associated with oxidation-reduction process were detected after 4 months of embryo development (mitochondrial benzaldehyde dehydrogenase, formate dehydrogenase and dehydroascorbate reductase) (Table 3). According to Fares et al. (2011), DHAR might constitute a common target for endogenously *S*-nitrosation in plants. In Brazilian pine dehydroascorbate reductase (DHAR) is more abundant in cell lines responsive to maturation promoters (ABA and osmotic agents) in comparison to blocked cell lines (dos Santos et al., 2016), and in our study, the cysteine residue (ERGDC^24^PFSQR) was *S*-nitrosated after 2 months of maturation in B2, and after 4 months of maturation in Y1 (Tables 3, 4). After 2 months of maturation, we detected in both cell lines an endogenously *S*-nitrosated mitochondrial benzaldehyde dehydrogenase (BALDH), (at Cys128 in cell line Y1 and at Cys345 in cell line B2) (Tables 3, 4). BALDH can oxidize benzaldehyde to benzoic acid (BA), an essential structural component for plant hormones, cofactors, defense compounds, and attractants for pollinators and seed dispersers (Widhalm and Dudareva, 2015). BA was found at relatively high concentrations in *Picea abies* embryos during the entire SE process (Vondrakova et al., 2018), suggesting a role of BALDH during somatic embryo formation. In general, taking into account that mild oxidative conditions are important during somatic embryo formation and that under stress conditions, antioxidant enzymes are affected by *S*-nitrosation, it is reasonable to suggest that redox signaling is actively mediating cellular process during somatic embryo maturation in cell line Y1 and probably interfering with PEMs differentiation in cell line B2.

#### S-Adenosylmethionine Cycle/Biosynthetic Process

Methionine adenosyltransferase (MAT) was detected as *in vitro S*-nitrosated during PEMs proliferation only in the B2 cell line (Table 2). MAT and Adenosylhomocysteinase are part of the methylMet cycle which provides activated methyl groups in transmethylation reactions (Yu et al., 2012). MAT (also known as S-adenosylmethionine synthetase, SAMS), catalyzes the biosynthesis of S-adenosylmethionine (AdoMet), a precursor for ethylene and polyamines biosynthesis in plants. In Arabidopsis, *S*-nitrosation of a recombinant MAT1 with GSNO provokes enzyme inhibition, which results in the reduction of the ethylene emission (Lindermayr et al., 2006; Jain et al., 2018). A differential accumulation of ethylene, polyamines, and AdoMet is considered essential during early somatic embryo differentiation in Brazilian pine (Jo et al., 2014). This result underlines a possible influence of *S*-nitrosation during PEMs differentiation in cell lines with contrasting embryogenic potential, and supports the importance of MAT as a biochemical marker of early somatic embryo development.

To summarize, endogenous SNO levels and GSNOR activity were determined during somatic embryogenesis in Brazilian pine. Furthermore, cysteine *S*-nitrosation site and *S*-nitrosated proteins (*in vivo* and *in vitro*) were identified by iodo-TMT labeling qualitative proteomics during PEMs proliferation and somatic embryo development. Notwithstanding the contrasting embryogenic potential in cell lines Y1 and B2, an overall increase in the content of endogenous SNO and GSNOR activity was observed during cell line cultivation in the medium supplemented with ABA and osmotic agents. *In vitro* and *in vivo S*-nitrosated proteins were associated with a large variety of biological processes, with some of them already described as important for somatic embryo formation (Class IV chitinase, pyruvate dehydrogenase E1 and dehydroascorbate reductase). To the best of our knowledge, this is the first time that protein *S*-nitrosation is demonstrated during somatic embryogenesis. By identifying new and previously described plant *S*-nitrosated proteins as differently present at distinct contexts of somatic embryogenesis, our data facilitates future studies focusing on elucidating how the activity and function of these proteins is affected by this PTM during somatic embryo differentiation.

## Data Availability Statement

The data presented in the study are deposited in the ProteomeXchange Consortium repository, accession number: PXD033177.

## Author Contributions

AB, GC, RZ, RS, and MR performed the experiments. RS, BM, LF, EF, and AW analyzed the data. AW conceived and coordinated the research. RS, LF, EF, and AW wrote the manuscript. All authors contributed to the article and approved the submitted version.

## Conflict of Interest

The authors declare that the research was conducted in the absence of any commercial or financial relationships that could be construed as a potential conflict of interest.

## Publisher’s Note

All claims expressed in this article are solely those of the authors and do not necessarily represent those of their affiliated organizations, or those of the publisher, the editors and the reviewers. Any product that may be evaluated in this article, or claim that may be made by its manufacturer, is not guaranteed or endorsed by the publisher.

## Footnotes

1
http://ekhidna2.biocenter.helsinki.fi

2
http://smart.embl.de

3
http://prosite.expasy.org/

4
http://www.ebi.ac.uk/interpro/

5
https://prosite.expasy.org/mydomains/

6
http://sno.biocuckoo.org/

7
http://app.aporc.org/iSNO-AAPair/

8
http://clavius.bc.edu/
