# Supplementary material for: Proteomic Analysis of S-Nitrosation Sites During Somatic Embryogenesis in Brazilian Pine, Araucaria angustifolia (Bertol.) Kuntze
Source: Front Plant Sci. 2022 Jun 30;13:902068. doi: 10.3389/fpls.2022.902068 (PMC9280032; doi:10.3389/fpls.2022.902068)
Supplement: Supplementary file 4 [file Data_Sheet_1.PDF]

Supplementary data S3. Comparison between *S*-nitrosated Cys sites computationally predicted using GPS SNO 1.0 or ISNO AAPAIR with Cys sites identified by iodo-TMT reagent in embryogenic cell lines of Brazilian pine.

**Software**	**Condition**	**Number of Cys-sites predicted *in silico***	**Number of Cys-sites equally identified by iodo-TMT and predicted *in silico***
GPS-SNO 1.0	In vivo *S*-nitrosated	14	4 (28.57%)
	In vivo + in vitro *S*-nitrosated	52	15 (28.84%)
ISO-AAPAIR	In vivo *S*-nitrosated	29	6 (20.68%)
	In vivo + in vitro *S*-nitrosated	89	18 (20.22%)
