# Supplementary material for: Proteomic Analysis of S-Nitrosation Sites During Somatic Embryogenesis in Brazilian Pine, Araucaria angustifolia (Bertol.) Kuntze
Source: Front Plant Sci. 2022 Jun 30;13:902068. doi: 10.3389/fpls.2022.902068 (PMC9280032; doi:10.3389/fpls.2022.902068)
Supplement: Supplementary file 5 [file Data_Sheet_2.PDF]

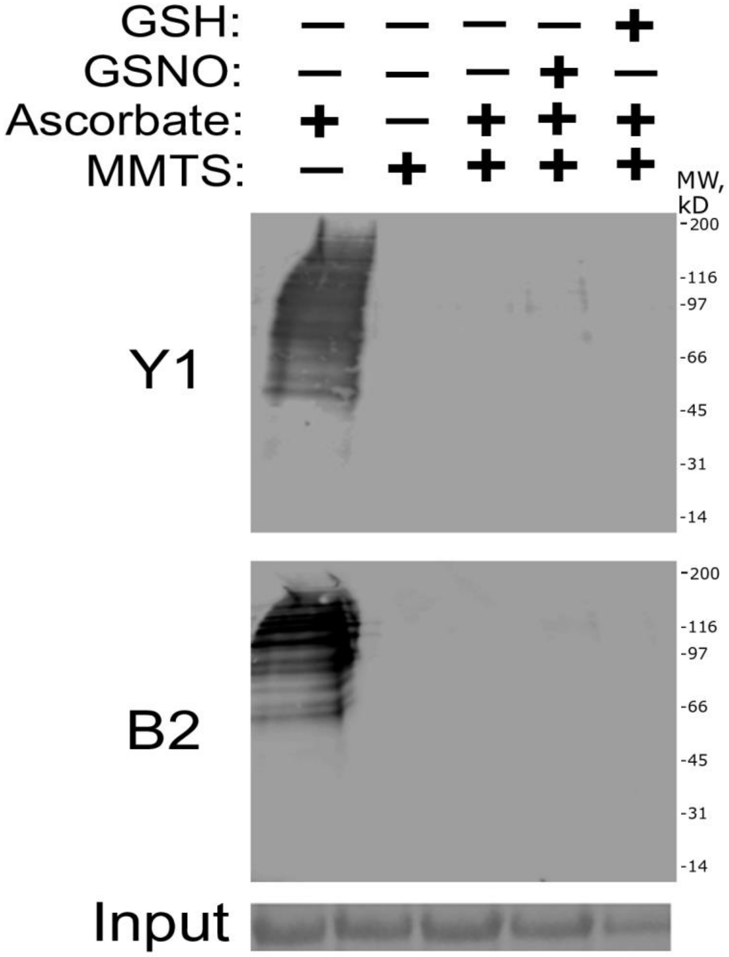

Supplemental Data S4. Representative Western blot demonstrating the detection of *in vivo* and *in vitro* S-nitrosated proteins in embryogenic cell lines of Brazilian pine using the iodo-TMT reagent. Cell lysates obtained after 14 days of PEMs proliferation were treated for 30 min with 500 µM GSNO, 500 µM GSH or ddH_2O and then labeled with iodo-TMT reagent. Proteins were separated with 12% SDS-PAGE, probed with anti-TMT antibody and detected using chemiluminescence method. The Ponceau S stained membrane is shown in the bottom of the figure (Input). 25 µg protein was loaded per lane. Two biological experiments were replicated. Y1, cell lines responsive to the formation of somatic embryos; B2, cell line blocked to the differentiation of somatic embryos.
