# Supplementary material for: Proteomic Analysis of S-Nitrosation Sites During Somatic Embryogenesis in Brazilian Pine, Araucaria angustifolia (Bertol.) Kuntze
Source: Front Plant Sci. 2022 Jun 30;13:902068. doi: 10.3389/fpls.2022.902068 (PMC9280032; doi:10.3389/fpls.2022.902068)
Supplement: Supplementary file 6 [file Data_Sheet_3.PDF]

Supplementary data S5. Putative S-nitrosated proteins and cysteines sites identified by iodo-TMT126 from GSNO-treated cell line Y1, after four months of cultivation on maturation medium MSG (Beckwar et al. 1989) supplemented with sucrose, sorbitol, ABA and solidified with gellan gum.

GO Biological process	Acession number	Protein name	Peptide sequence
		After two months of maturation	
**Translation-related process**	A9NTN5	KH type-2 domain-containing protein	GLC^{97}AIAQAESLR
	A0A200R162	Translation elongation factor EFG	RQESVEDVPC^{448}GNTVAMV
	A0A0C9RRN5	Eukaryotic translation initiation factor 5A	C^{58}HFVGIDIFNGK
	A0A0C9RYZ2	60S ribosomal protein	ANTFVIDC^{27}GKPVEDK
	A0A1U8AGL3	40S Ribosomal protein S11-beta	C^{59}PFTGNVSIR
	A0A0D6QYG9	40S ribosomal protein SA	NC^{31}DFQMER
	A0A0D6R1L5	60S ribosomal protein L11	IAC^{68}FVTVR
	A0A0D6R6S4	WD_REPEATS_REGION domain-containing protein	VWNLTNC^{184}K
	A0A0C9S3L3	Elongation factor 1-alpha	YYC^{87}TVIDAPGHR
	A0A0D6QX01	Ribosomal protein 1	DDPSKPC^{41}K
	A0A0D6R480	Ribosomal protein L2	SIPEGAIVC^{114}NVEQHAGDR
	A0A0D6QUM0	60S ribosomal protein L18	AGGEC^{55}LTFDQLALR
**Proteolysis**	A0A2G2XGY5	Subtilisin-like protease	GVFVSC^{305}SAGNSGPGGDTVS
	A0A0D6R551	Proteasome subunit alpha	IIEINPYLLGTMAGGAADC^{107}QFWHR
**Defense response**	Q9SNX7	Putative intracellular pathogenesis-related protein	KIEAYLLSNPALYC^{161}
**Carbohydrate metabolic process**	A0A0D6QYH9	UTP--glucose-1-phosphate uridylyltransferase	LNGGLGTTMGC^{101}TGPK
	A0A0D6R7Z3	2,3-bisphosphoglycerate-independent phosphoglycerate mutase	IQILTSHTC^{503}SPVPIAIGGPGLK
	A0A3S3PBW7	Endo-beta-N-acetylglucosaminidase	QVLLSAAPQC^{190}PYPDAHLGR
	A0A0D6QYJ0	Pyruvate dehydrogenase	ILTTDSFDWTC^{120}SDETPAR
	Q5NTA4	Class IV chitinase	NPPINYC^{182}DSSNK
**Oxidation-reduction process**	C7A2A0	Mitochondrial benzaldehyde dehydrogenase	VGTVWINC^{497}FDTFDAAIPFGGYK
	A0A089MX36	Dehydroascorbate reductase	ERGDC^{24}PFSQR
	A0A0C9RI13	S-(hydroxymethyl)glutathione dehydrogenase	GVTEVQPGDHVIPCYQAEC^{99}R

	A9NV09	Formate dehydrogenase, mitochondrial	GHQYIVTDDKEGPNC^{84}ELER
	A0A0D6R8F1	Succinate--CoA ligase [ADP-forming] subunit beta, mitochondrial	C^{361}DIIASGIVNAAK
	A0A0D6R4M7	Cytosolic isocitrate dehydrogenase	NILNGTVFREPILC^{116}K
**ATP biosynthetic process**	A0A0C9SAE7	ATP synthase subunit beta	C^{305}ALVYGQMNEPPGAR
	A0A0C9S3G1	Pyruvate kinase	VVDSPCC^{570}LVTGEYGWTANMER
**S-adenosylmethionine cycle**	A0A0C9RQC5	Adenosylhomocysteinase	GETLQEYWWC^{240}TER
**Protein folding**	A0A4D6N4G6	Chaperonin GroEL	AIFAEGC^{136}K
	A0A0D6R5L9	Peptidyl-prolyl cis-trans isomerase	GPNTNGSQFFLC^{212}TVK
**Amino acid metabolic process**	A0A0C9SA76	Fumarylacetoacetase	NC^{143}GIIFR
**Cellular process**	A0A6A2XTS2	Epidermis-specific secreted glycoprotein EP1	C^{430}FLTQSLDTLQQLGNTK
	A0A251RRV5	Expansin-like protein	NGGGC^{86}SAC^{89}YQIR
	A0A0D6R8S3	Ricin B, lectin domain-containing protein	VYC^{115}EANPDFFLAAR
		After four months of maturation	
**Translation-related process**	A0A1U8AGL3	40S Ribosomal protein S11-beta	C^{59}PFTGNVSIR
	A0A0D6R6S4	WD_REPEATS_REGION domain-containing protein	VWNLTNC^{184}K
	A0A0D6QX01	Ribosomal protein 1	DDPSKPC^{41}K
**ATP biosynthetic process**	A0A0C9SAE7	ATP synthase subunit beta	C^{305}ALVYGQMNEPPGAR
**Oxidation-reduction process**	A0A089MX36	Dehydroascorbate reductase	ERGDC^{24}PFSQR
**Defense response**	Q9SNX7	Putative intracellular pathogenesis-related protein	ERVDELDENNFC^{82}YK
**Carbohydrate metabolic process**	A0A3S3PBW7	Endo-beta-N-acetylglucosaminidase	QVLLSAAPQC^{190}PYPDAHLGR
	Q5NTA4	Class IV chitinase	NPPINYC^{182}DSSNK
