# Supplementary material for: Proteomic Analysis of S-Nitrosation Sites During Somatic Embryogenesis in Brazilian Pine, Araucaria angustifolia (Bertol.) Kuntze
Source: Front Plant Sci. 2022 Jun 30;13:902068. doi: 10.3389/fpls.2022.902068 (PMC9280032; doi:10.3389/fpls.2022.902068)
Supplement: Supplementary file 7 [file Data_Sheet_4.PDF]

Supplementary data S6. Putative *S*-nitrosated proteins and cysteines sites identified by iodo-TMT reagent from GSNO-treated cell line B2, after four months of cultivation on maturation medium MSG (Beckwar et al. 1989) supplemented with sucrose, sorbitol, ABA and solidified with gellan gum.

GO Biological process	Acession number	Protein name	Peptide sequence
		After two months of maturation	
Translation-related process	A0A200R162	Translation elongation factor EFG	$NTGSPTC^{267}K$
	A0A1U8AGL3	40S Ribosomal protein S11-beta	$C^{59}PFTGNVSIR$
	A0A0D6QYG9	40S ribosomal protein SA	$NC^{31}DFQMER$
	A0A0D6R1L5	60S ribosomal protein L11	$IAC^{68}FVTVR$
	A0A0C9S3L3	Elongation factor 1-alpha	$YYC^{87}TVIDAPGHR$
Carbohydrate metabolic process	A0A0D6R7Z3	2,3-bisphosphoglycerate-independent phosphoglycerate mutase	$IQILTSHTC^{503}SPVPIAIGGPGLK$
	A0A0D6QYS8	Pyruvate dehydrogenase E1 component subunit alpha, mitochondrial	$DC^{119}IITAYR$
	Q5NTA4	Class IV chitinase	$NPPINYC^{182}DSSNK$
Protein folding	A0A4D6N4G6	Chaperonin GroEL	$C^{245}ELENPLILIHEK$
	A0A0D6R5L9	Peptidyl-prolyl cis-trans isomerase	$C^{103}FFDVEIGGVPAGR$
Defense response	Q9SNX7	Putative intracellular pathogenesis-related protein	$ERVDELDENNFC^{82}YK$
Oxidation-reduction process	C7A2A0	Mitochondrial benzaldehyde dehydrogenase	$SPFIVC^{320}EDADVDK$
	A0A0D6R325	Protein disulfide-isomerase	$AEGVPC^{330}ILIQDTENR$
	A0A0D6R8F1	Succinate--CoA ligase [ADP-forming] subunit beta, mitochondrial	$C^{361}DIIASGIVNAAK$
	A9NV09	Formate dehydrogenase, mitochondrial	$GHQYIVTDDKEGPNC^{84}ELER$
Amino acid metabolic process	A0A0C9SA76	Fumarylacetoacetase	$NC^{143}GIIFR$
Cellular process	A0A251RRV5	Expansin-like protein	$NGGGC^{86}SACYQIR$
	A0A6A2XTS2	Epidermis-specific secreted glycoprotein EP1	$C^{430}FLTQSLDTLQQLGNTK$
		After four months of maturation	
Carbohydrate metabolic process	Q5NTA4	Class IV chitinase	$NPPINYC^{182}DSSNK$
