# Supplementary material for: Proteomic Analysis of S-Nitrosation Sites During Somatic Embryogenesis in Brazilian Pine, Araucaria angustifolia (Bertol.) Kuntze
Source: Front Plant Sci. 2022 Jun 30;13:902068. doi: 10.3389/fpls.2022.902068 (PMC9280032; doi:10.3389/fpls.2022.902068)
Supplement: Supplementary file 8 [file Data_Sheet_5.PDF]

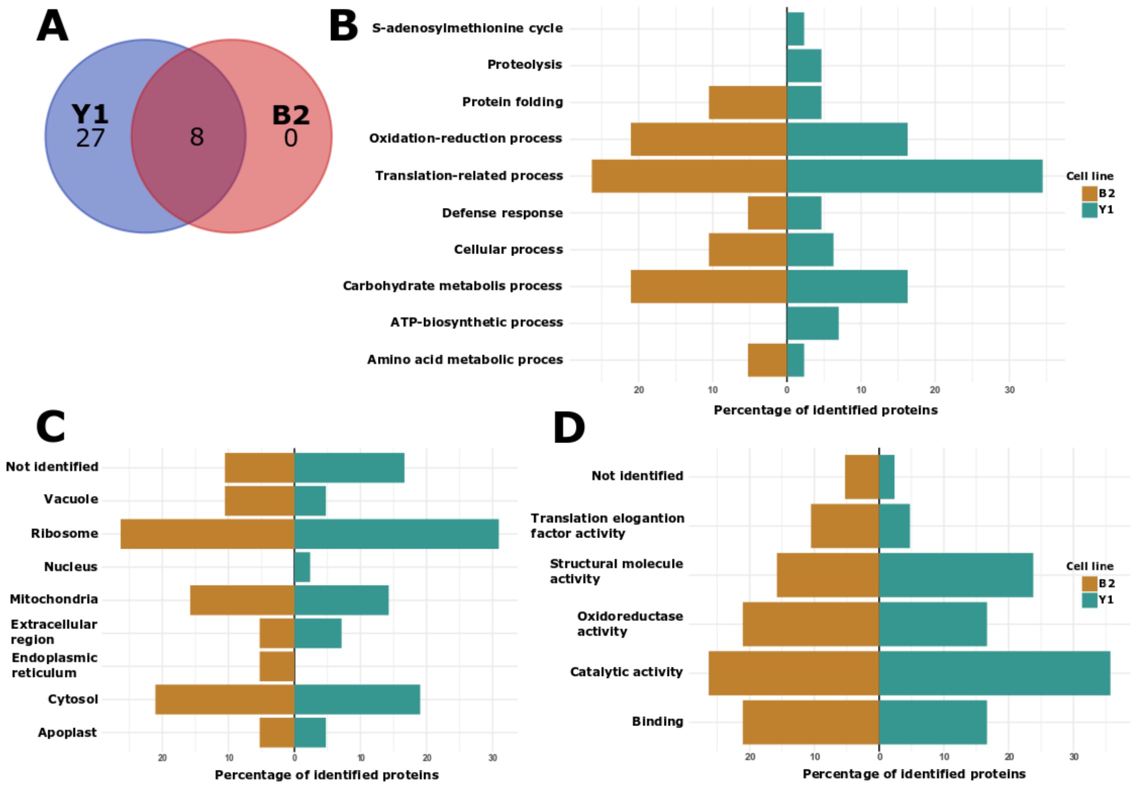

Supplementary data S7. Characterization of *in vitro* S-nitrosated proteins detected during cultivation of cell lines Y1 and B2 of Brazilian pine in medium culture supplemented with maturation promoters (osmotic agents and ABA). (A) VENN diagram depicting the number of identified proteins in cell lines Y1 and B2 after four months of maturation; (B) Biological process classification in GO analysis of proteins detected in cell lines Y1 and B2 after four months of maturation; (C) Cellular component and (D) Molecular function.
