# Supplementary material for: Proteomic Analysis of S-Nitrosation Sites During Somatic Embryogenesis in Brazilian Pine, Araucaria angustifolia (Bertol.) Kuntze
Source: Front Plant Sci. 2022 Jun 30;13:902068. doi: 10.3389/fpls.2022.902068 (PMC9280032; doi:10.3389/fpls.2022.902068)
Supplement: Supplementary file 9 [file Data_Sheet_6.PDF]

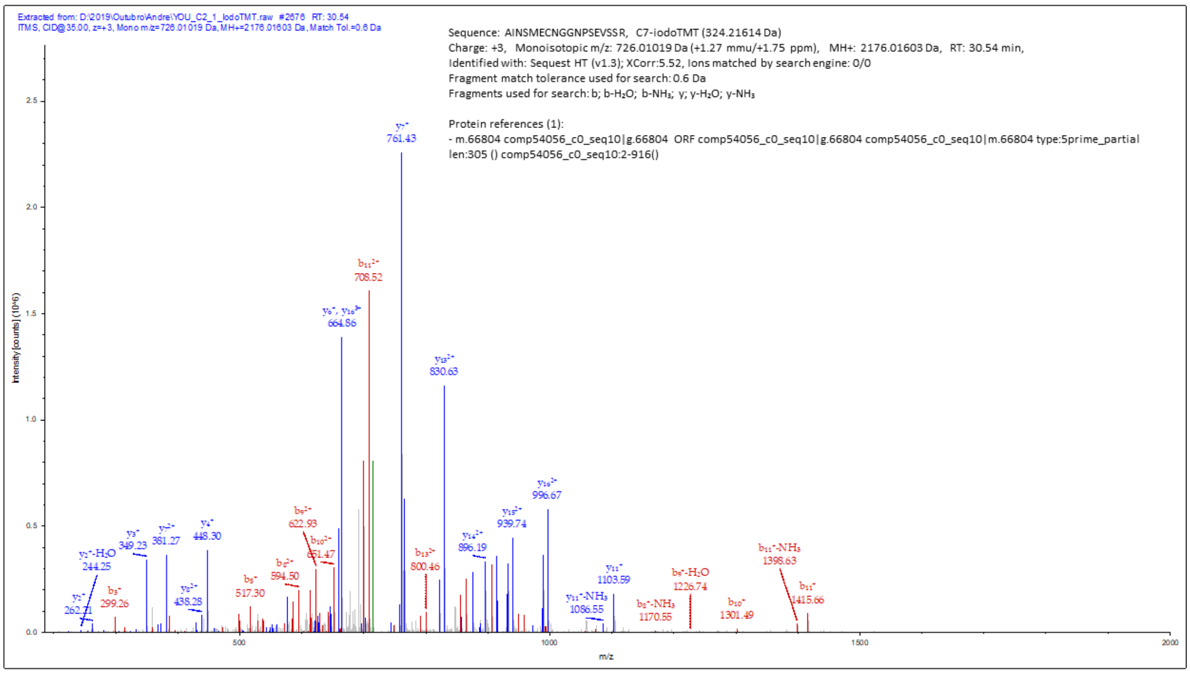

Figure S8.A- Representative MS/MS spectra of *S*-nitrosylated peptide AINSMECNGGNPSEVSSR, C7-iodoTMT (324.21614 Da) from Class IV chitinase.

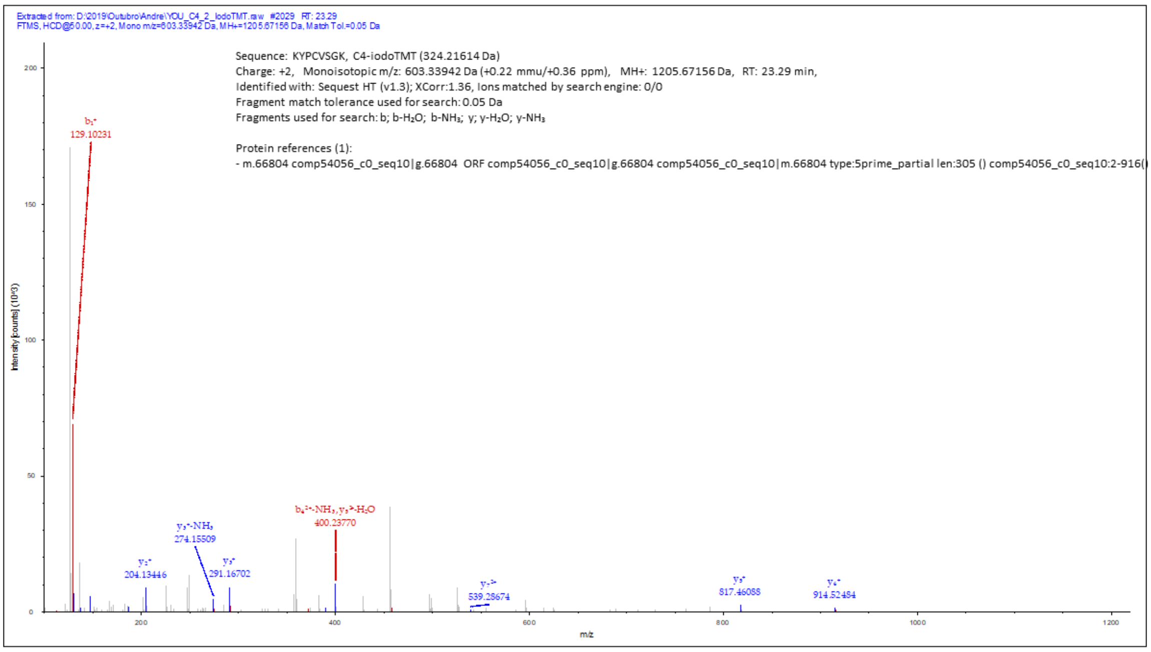

Figure S8.B- Representative MS/MS spectra of *S*-nitrosylated peptide KYPCVSGK, C4-iodoTMT (324.21614 Da) from Class IV chitinase.

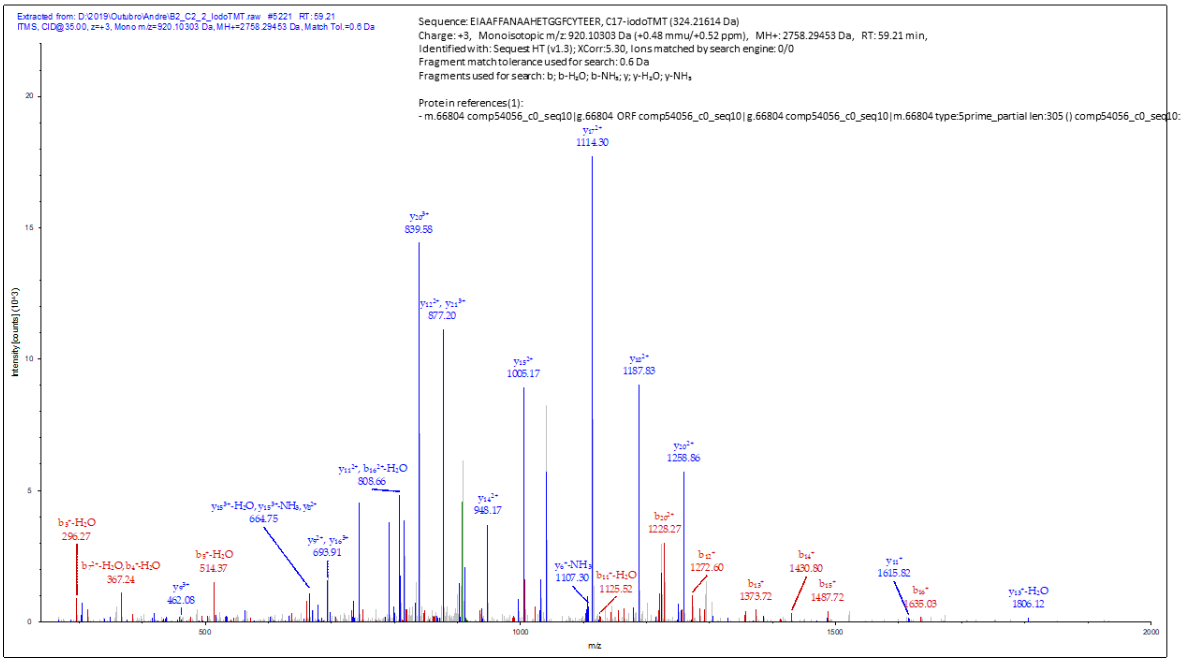

Figure S8.C- Representative MS/MS spectra of *S*-nitrosylated peptide EIAAFFANAAHETGGFCYTEER, C17-iodoTMT (324.21614 Da) from Class IV chitinase.

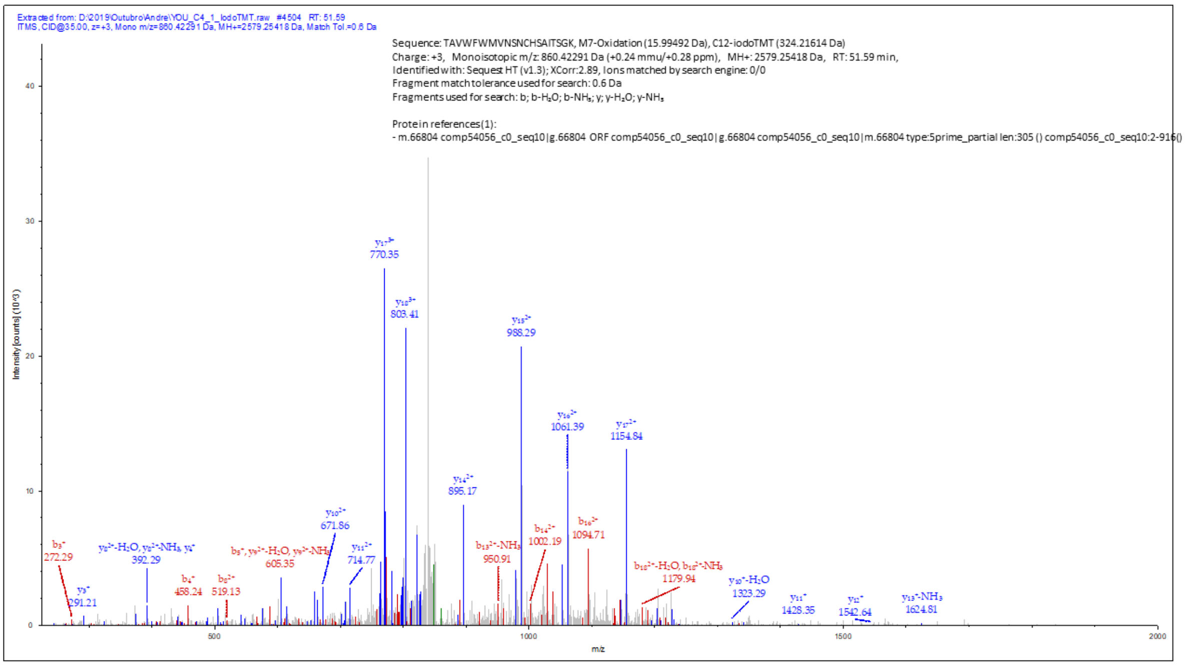

Figure S8.D- Representative MS/MS spectra of *S*-nitrosylated peptide TAVWFWMVNSNCHSAITSGK, C12-iodoTMT (324.21614 Da) from Class IV chitinase.
